# Cemented versus Uncemented Oxford Unicompartmental Knee Arthroplasty: Is There a Difference?

**DOI:** 10.1155/2013/245915

**Published:** 2013-12-09

**Authors:** Burak Akan, Dogac Karaguven, Berk Guclu, Tugrul Yildirim, Alper Kaya, Mehmet Armangil, Ilker Cetin

**Affiliations:** ^1^Department of Orthopedics and Traumatology, Ufuk University Faculty of Medicine, Ankara, Turkey; ^2^Dikmen C Parkpinar Evleri, 9/B No 28 Keklikpinari Cankaya, 06420 Ankara, Turkey; ^3^Department of Orthopedics and Traumatology, Ankara University Faculty of Medicine, Ankara, Turkey

## Abstract

*Purpose*. The use of uncemented unicompartmental knee prostheses has recently increased. However, few studies on the outcomes of uncemented unicompartmental knee prostheses have been performed. The purpose of this study was to compare the outcomes of cemented and uncemented Oxford unicompartmental knee arthroplasty. *Materials and Methods*. This retrospective observational study evaluated the clinical and radiological outcomes of 263 medial Oxford unicompartmental prostheses (141 cemented, 122 uncemented) implanted in 235 patients. The mean follow-up was 42 months in the cemented group and 30 months in the uncemented group. *Results*. At the last follow-up, there were no significant differences in the clinical results or survival rates between the two groups. However, the operation time in the uncemented unicompartmental knee arthroplasty group was shorter than that in the cemented unicompartmental knee arthroplasty group. In addition, the cost of uncemented arthroplasty was greater. *Conclusion*. Despite the successful midterm results in the uncemented unicompartmental knee arthroplasty group, a longer follow-up period is required to determine the best fixation mode.

## 1. Introduction

Unicompartmental knee replacement arthroplasty (UKA) has been a popular treatment of osteoarthritis since the 1970s. Initial reports showed high failure rates in short-term follow-ups [1]. Because of these high failure rates and instrumentation problems, the use of these implants decreased in the 1990s. However, during the last 20 years, UKA has become a well-established treatment method for unicompartmental osteoarthritis of the knee. Recent reports have described success rates of 90% or higher at a minimum 10-year follow-up [2, 3]. These higher success rates have been attributed to better surgical techniques, new implant designs, improved instrumentation, and careful patient selection [4]. With the improvements in surgical techniques and instruments, this procedure has many advantages over total knee replacement such as a smaller incision, less soft tissue injury, preservation of bone stock, preservation of normal knee kinematics, less morbidity because of minimal postoperative blood loss, lower infection rate, shortened hospital stay, and rapid recovery [5]. However, controversy on the validity and durability of UKA remains. Although UKA is associated with better clinical results than total knee replacement arthroplasty (TKA), registry data show higher revision rates [6]. On the other hand, Goodfellow et al. reported that the revision rate is a poor and misleading outcome and questioned its use in comparing UKA and TKA [7].

Most UKA designs use cement to fix the components to the bone. Disadvantages of cemented UKA prostheses are aseptic loosening, third-body particles in the joints, and extended surgical times. A porous-coated UKA design is an alternative to cemented fixation [8]. In addition to inducing bone growth, it also provides more reliable fixation, especially in younger patients. Some studies have reported excellent results of uncemented TKA, and these results were similar to those of cemented TKA [9, 10]. However, registry data and meta-analyses show superior results and durability in cemented TKA over uncemented TKA [11]. The efficacy of the uncemented design in UKA remains unclear. A few encouraging reports of cemented and uncemented UKAs have been published, but few direct comparisons of these two methods have been made [12]. In this study, we retrospectively evaluated the early clinical and functional outcomes of cemented and uncemented Oxford Phase 3 UKAs.

## 2. Materials and Methods

From August 2008 to May 2011, a total of 235 patients (263 knees) underwent UKAs with mobile-bearing Oxford unicompartmental knee prostheses (Biomet UK Ltd., Bridgend, UK). There were 141 cemented and 122 uncemented UKAs. All patients had anteromedial osteoarthritis and conformed to the indications described by Goodfellow et al.: patients with medial osteoarthritis, a correctable varus deformity, an intact anterior cruciate ligament and collateral ligaments, absence of degenerative findings in the lateral compartment of the knee on standing radiographs, and femoral flexion deformity of less than 15° [13]. Osteoarthritis of the patellofemoral joint, obesity, old age, and high activity level were not considered to be contraindications to this procedure, unlike the Kozinn and Scott criteria [14, 15]. The final decision to proceed with unicompartmental arthroplasty was made at the time of surgery. If arthritis was found in the lateral compartment or if there was no anterior cruciate ligament, then the operation was converted to total knee arthroplasty. One patient in the uncemented group had undergone arthroscopic anterior cruciate ligament reconstruction 1 year before the index surgery.

The two groups were well matched for age. The mean age was 64.2 years in the cemented group (range, 42–84 years) and 64.9 years in the uncemented group (range, 35–79 years). There were 20 male and 100 female patients in the cemented group and 11 male and 104 female patients in the uncemented group. The mean body mass index was 29.8 (range, 18.5–41.8) in the cemented group and 28.6 (range, 18.8–38.8) in the uncemented group.

The use of cementless components began in 2009; previous to this time, they were not available in the market. Because the manufacturers introduced cementless components during the study period, all surgeons started to perform cementless UKA. In contrast to cemented prostheses, the cementless UKA has a layer of porous titanium with calcium hydroxyapatite under its components and some mechanical modifications to allow for cementless fixation.

All of the surgical procedures were performed by four surgeons (Ilker Cetin, Alper Kaya, Berk Guclu, and Burak Akan) or under their supervision. All operations were performed under tourniquet control, via a short paramedian skin incision, following a medial parapatellar arthrotomy. The cemented and uncemented surgical techniques were identical with the exception of application of the last components.

Early active movement and full weight-bearing were allowed postoperatively, and thromboprophylaxis was prescribed for all patients. All patients were routinely followed up at 6 weeks, 3 months, 6 months, and 1 year postoperatively. The Knee Society objective and functional scores and the Oxford Knee score were used to assess the clinical outcomes.

SSPS ver. 15.0 (SPSS Inc., Chicago, IL, USA) was used for the statistical analyses. Quantitative variables are shown as means, standard deviations, medians, numbers, and percentages. Normality of continuous variables was evaluated by the Kolmogorov-Smirnov test. An independent-samples *t*-test was used to determine the difference between the two groups when parametric test assumptions were satisfied. The groups were compared by the Mann-Whitney *U* test when parametric test assumptions were not satisfied. The chi-square test was used to compare qualitative variables. Differences between the preoperative and postoperative values were evaluated within each group and between the two groups using a variance analysis in repeated measurements. The level of statistical significance was set at *P* = 0.05.

## 3. Results

A total of 235 patients (263 knees) were included in the study. The mean follow-up was 42 months (range, 24–52 months) in the cemented group and 30 months (range, 24–36 months) in the uncemented group. The mean preoperative and postoperative range of motion of the cemented cases were 114° (range, 100°–130°) and 128° (range, 115°–145°), respectively. The mean preoperative and postoperative range of motion of the uncemented cases were 119° (range, 105°–132°) and 133° (range, 120°–150°), respectively. There were no statistically significant differences in the demographic data (Table 1).

The mean preoperative Oxford Knee score in the cemented group was 15.4 points (range, 9–23 points), and that in the uncemented group was 20.9 points (range, 12–29 points). The mean postoperative Oxford knee score in the cemented group was 39.3 points (range, 29–47 points), and that in the uncemented group was 41.1 points (range, 34–47 points). There were no statistically significant differences in the outcomes between the groups (*P* = 0.452). The mean preoperative Knee Society score for cemented UKA improved from 43.7 points (range, 32–55 points) to 87.7 points (range, 71–100 points) at the last follow-up. The mean preoperative Knee Society score for uncemented UKA increased from 43.8 points (range, 32–57 points) to 87.6 points (range, 72–100 points) at the last follow-up. There were no significant intergroup differences in the knee scores (*P* = 0.897, *P* = 0.721). The mean preoperative functional score for cemented UKA increased from 58.4 points (range, 42–69 points) to 88.2 points (range, 72–100 points). The mean preoperative and postoperative functional scores for uncemented UKA were 59.0 points (range, 40–76 points) and 90.2 points (range, 68–100 points), respectively. There were no significant intergroup differences in the functional scores (*P* = 0.841, *P* = 0.624).

The revision rate was 7.09% (10 knees) in the cemented group and 4.91% (6 knees) in the uncemented group (Table 2). There was no statistically significant difference in the revision rate (*P* = 0.155). Mobile-bearing dislocation occurred in four patients in the cemented group and three patients in the uncemented group. One patient was revised to a mobile bearing that was one size thicker; the other patients were converted to TKA. Four patients in the cemented group and three patients in the uncemented group underwent TKA because of persistent unexplained pain. Lateral arthritis occurred in one patient in the cemented group. This patient underwent TKA. In the uncemented group, one medial tibial plateau fracture and one medial femoral condyle fracture occurred. There was one medial tibial plateau fracture in the cemented group. The tibial plateau fractures were treated with TKA, and the femoral condyle fracture was treated with percutaneous cannulated screws.

The mean operation time of cemented UKA was 45.3 minutes (range, 30–58 minutes), and the mean operation time of uncemented UKA was 36.1 minutes (range, 27–47 minutes). There was a significant difference between these operation times (*P* < 0.001). The mean follow-up was 42 months in the cemented group and 30 months in the uncemented group. There is a significant statistical difference in the follow-up period between the two groups (*P* < 0.001).

## 4. Discussion 

The most important finding of the present study was that uncemented Oxford UKA provided good clinical and functional patient outcomes similar to those obtained with cemented prostheses. The medial tibiofemoral compartment is the most common of the three knee compartments affected by degenerative joint disease [16]. The predictable advantages of UKA over proximal tibial osteotomy are quicker recovery, relief of pain, and much better long-term results. In appropriately selected patients described by designers, UKA has many advantages over total arthroplasty including more satisfactory physiological functions, quicker recovery, and easier revision in cases of failure [5, 17]. Although unicompartmental knee arthroplasty is not a new procedure, the use of and interest in this technique have increased in Turkey during the last 5 years. The main reasons for the increased popularity of unicompartmental knee arthroplasty are the introduction of minimally invasive surgical techniques with modified surgical equipment and the publication of excellent mid- and long-term results [16, 18]. Among the many controversies concerning UKA is the question of which design concept is associated with the most simple, reproducible surgical technique and optimal long-term results. Other specific controversies include fixed-bearing versus mobile-bearing designs, onlay versus inlay designs, the use of robotics and customization, and whether total knee arthroplasty represents a better treatment solution. However, there is no controversy between cemented and uncemented fixation.

**Figure 1:**
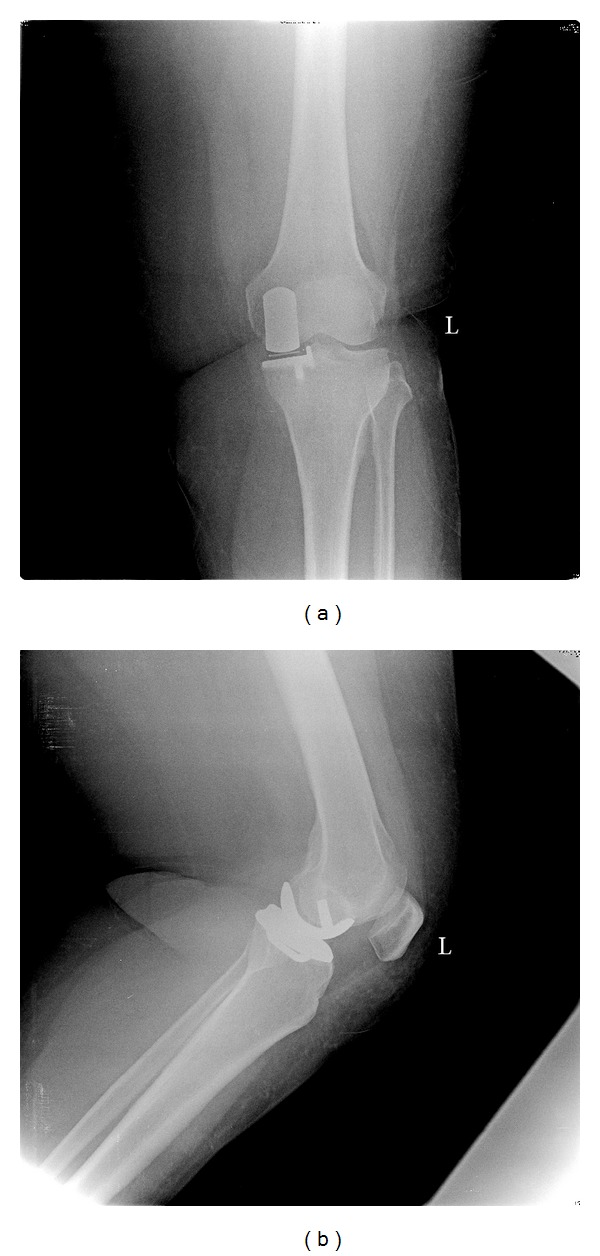
(a) A 56-year-old female patient that underwent cemented Oxford UKA, anterior-posterior view. (b) Lateral view.

**Figure 2:**
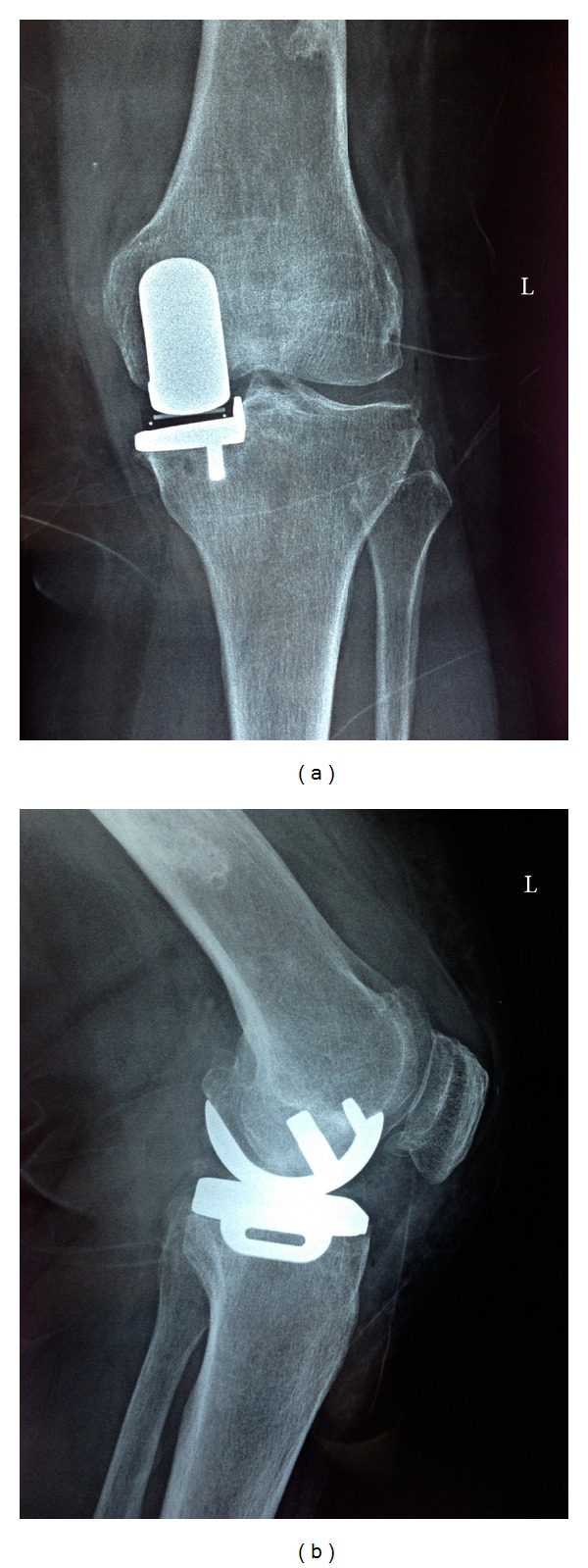
(a) A 51-year-old female patient that underwent uncemented Oxford UKA, anterior-posterior view. (b) Lateral view.

The results of cemented and uncemented total knee replacements have been examined in many previous studies, but ongoing debate about the fixation type remains. The Oxford UKA prosthesis (Biomet UK Ltd., Bridgend, UK), which has been used as a cemented implant for many years, required relatively few modifications of its components to satisfy the perceived requirements for uncemented fixation (Figures 1 and 2). Many surgeons prefer using cemented knee prostheses, although there is a considerable debate regarding the possible benefits of using cementless fixation in joint replacement surgery. These debates include bone stock preservation, avoidance of cementation complications, ease of revision surgery, and improved long-term survival of the implant. Cementation errors and third bodies can cause pain, impingement, dislocation and wear of the bearing, and unnecessary revision because of misunderstandings of the significance of radiolucency, as in aseptic loosening. In minimally invasive surgical techniques, it is difficult to clean the cementation residuals from the posterior aspect, and it is possible that these residuals can prevent mobility of the bearings.

Another benefit of using an uncemented prosthesis is the shortened operation time. In our study, the operation time was significantly shorter in the uncemented group. Shortening the operation time reduces the infection rate and tourniquet side effects and improves operation room productivity [19].

However, whether cementless implants improve long-term patient survival is unclear. Few studies have researched uncemented unicompartmental knee arthroplasty. Pandit et al. reported no differences in patient outcomes between cemented and uncemented fixation, and uncemented fixation was associated with reduced radiolucency after 1 year [8]. This radiolucency has been implicated in aseptic failure, particularly with persistent unexplained pain in surgeries performed by inexperienced surgeons. Using an uncemented prosthesis may help to avoid unnecessary revision surgeries. On the other hand, the cost of uncemented UKA is approximately 450 Euros more than the cost of cemented UKA in the UK market, and this may be a disadvantage of uncemented arthroplasty because no differences were noted in the clinical and functional results between the groups. The costs of uncemented and cemented UKA are very similar in Turkey; thus, we have improved accessibility to uncemented designs compared with other countries.

The rate of revision to TKA was not significantly different between the cemented and uncemented UKA groups in our study. The most common reasons for revision were unexplained pain and insert dislocation. These failures appeared more often than in independent series, but their incidence was similar to that in designer series [20, 21]. Some of our cases showing physiological radiolucency without pain were not considered to involve component loosening, and they were not revised to UKA. According to designers, physiological radiolucencies are generally 1 mm or less, are surrounded by a sclerotic margin, develop in the first year, and remain static; however, pathologic radiolucencies are wide and do not have a sclerotic margin [22]. It was reported that the incidence of radiolucent lines associated with uncemented Oxford medial unicompartmental knee replacements was significantly lower than that associated with cemented UKA [23]. We found no pathologic radiolucencies in either the cemented or uncemented UKA group.

Many surgeons tend to believe that UKA revision is more beneficial for patients with unexplained pain. Unicompartmental implants are reportedly more susceptible to revision, especially in patients with unexplained pain [7]. We experienced six patients with unexplained pain for whom initial UKA was converted to TKA. Causes of pain such as component loosening, periprosthetic infection, component malpositioning, and spine and hip diseases were all excluded before the revision surgery. The average interval from the primary UKA to the revision TKA for patients with unexplained pain was 15 months (range, 9–22 months). It is recommended to wait at least 2 years after surgery for pain relief because of bone remodeling [24].

We experienced both anterior and posterior mobile-bearing dislocations caused by retained osteophytes or imbalanced flexion-extension gaps. Individuals in Turkey require high degrees of knee flexion for religious and social reasons. Lim et al. reported that bearing dislocations occur more commonly in Asian than in Western cultures because of these demands [25]. Another cause of failure, lateral osteoarthritis, appeared in the early period in one patient.

There were two tibial periprosthetic fractures in cemented and uncemented UKA that were recognized during the postoperative follow-up period. These fractures occurred during daily life weight-bearing activities with minor trauma. Both were treated with standard TKA. Periprosthetic tibial fracture is an uncommon complication of cemented UKA and is related to technical errors. Seeger et al. reported that patients with an extended sagittal bone cut, especially those treated with cementless UKA, are at higher risk for periprosthetic tibial fracture [26]. Although a greater impaction force was applied to uncemented tibial components, there were no differences in the tibial periprosthetic fracture rates among our cases. There was one femoral condyle fracture in the uncemented UKA group that was treated with closed reduction and percutaneous fixation [27].

Our study shows that uncemented UKA provides good clinical and functional outcomes similar to those of cemented UKA. It has a similar complication rate and shorter operation time than cemented UKA. Unlike uncemented TKA, in countries where uncemented UKA prosthesis costs are similar to those of cemented prostheses, we suggest the use of uncemented UKA.

## 5. Conclusions

No clinical difference was observed between cemented and uncemented unicondylar knee prostheses. Uncemented UKA is as safe as cemented UKA. A longer follow-up period is required to determine the best fixation mode.

## Figures and Tables

**Table 1:** Demographical data.

	Cemented group (*n* = 141)	Uncemented group (*n* = 122)	*P * value
Age (years)	64.2	64.9	*P* = 0.746
Weight (kg)	74 ± 4.2	77 ± 5.5	*P* = 0.731
Height (cm)	158 ± 9.2	166 ± 10.0	*P* = 0.294
Body mass index (kg/cm^2^)	29.8 ± 11.2	28.6 ± 9.7	*P* = 0.771
Gender (female/male)	100/20	104/11	*P* = 0.213
Duration of surgery (min)	45.3 ± 12.1	36.1 ± 11.4	*P* < 0.001
Revision rate (%)	7.09%	4.91%	*P* = 0.153
Preoperative Oxford Knee score	15.4 ± 5.8	20.9 ± 6.2	*P* = 0.614
Postoperative Oxford Knee score	39.3 ± 7.6	41.1 ± 6.0	*P* = 0.452
Preoperative Knee Society score	43.7 ± 8.9	43.8 ± 9.2	*P* = 0.897
Postoperative Knee Society score	87.7 ± 10.5	87.6 ± 10.2	*P* = 0.721
Preoperative functional score	58.4 ± 7.8	59.0 ± 11.6	*P* = 0.841
Postoperative functional score	88.2 ± 8.1	90.2 ± 6.5	*P* = 0.624

**Table 2:** Reasons for revision.

	Cemented Oxford unicompartmental	Uncemented Oxford unicompartmental
Unexplained pain	4	2
Mobile-bearing dislocation	4	3
Lateral osteoarthritis	1	0
Tibial plateau fracture	1	1

Total	10	6

## Abbreviations

UKA: Unicompartmental knee arthroplasty
TKA: Total knee arthoplasty.
